# D-line Approach for Safe Laparoscopic Cholecystectomy: Initial Experience

**DOI:** 10.7759/cureus.45003

**Published:** 2023-09-10

**Authors:** Amr Badawy, Ibrahim Fathi, Tarek Sabra

**Affiliations:** 1 General Surgery, Faculty of Medicine, Alexandria University, Alexandria, EGY; 2 Pediatric Surgery, Faculty of Medicine, Assuit University, Assuit, EGY

**Keywords:** critical view of safety, difficulty, laparoscopic, cholecystectomy, diagonal line, segment iv

## Abstract

Introduction

The critical view of safety is an important concept for safe laparoscopic cholecystectomy. However, no standard step-by-step approach for achieving the critical view of safety has been established until now. Therefore, this study aims to evaluate a new approach for achieving the critical view of safety using the diagonal line of liver segment IV as an anatomical landmark.

Patients and methods

In this prospective non-randomized study, patients (n= 112) who underwent laparoscopic cholecystectomy for symptomatic cholelithiasis were included. The first 47 patients underwent laparoscopic cholecystectomy using the diagonal line approach (DLC group) whereas, the next 65 patients underwent laparoscopic cholecystectomy using the conventional method (CLC group).

Results

No significant difference between both groups regarding the preoperative characteristics, operative time, and intraoperative blood loss. Laparoscopic subtotal cholecystectomy was performed more in the DLC group (6% vs 0%, p= 0.07). Whereas, in the CLC group, there was a tendency towards conversion to open cholecystectomy in difficult cases (6% vs 2%, p= 0.40). No intra- or postoperative complications occurred in either group.

Conclusion

The diagonal line approach is a feasible and useful step-by-step technique to achieve the critical view of safety in laparoscopic cholecystectomy and enables surgeons to perform safe laparoscopic subtotal cholecystectomy in difficult cases.

## Introduction

Laparoscopic cholecystectomy (LC) is the gold standard treatment for symptomatic cholelithiasis and one of the most frequent laparoscopic procedures performed worldwide. Since the first LC in 1987, the number of performed LC procedures increased dramatically as well as the incidence of bile duct injury (BDI) [1,2] which reached 0.3-0.8% in comparison to 0.2% in open cholecystectomy [3]. BDI is mostly caused by the misidentification of the biliary structures, which gave rise to the concept of critical view of safety (CVS) by Strasberg et al in 1995[4]*.* CVS is used to safely identify the cystic structures through clearance of all fat and fibrous tissues in the Calot's triangle, separation of the lower third of the gallbladder (GB) from the liver bed, and confirmation of only two structures passing into the GB. However, this approach is sometimes very challenging to achieve in difficult LCs with frozen Calot’s triangle [1].

Tokyo guidelines in 2018 recommended the use of an imaginary line between the base of segment IV and Rouviere’s sulcus (RS) as an anatomical landmark to dissect the GB above that line in case of difficult LCs [5]. However, RS is not visible in 25% of cases, and sometimes the base of segment IV is obscured by dense adhesions [6]. That is why Kitamura et al [1] proposed the diagonal line (D-line) of liver segment IV as a new anatomical landmark to achieve CVS, especially in the case of a difficult LC. Therefore, this study aimed to externally validate this new approach for LCs.

## Materials and methods

Study population

In this prospective non-randomized study, patients (n=112) with symptomatic cholelithiasis who were indicated for LC were included. In the first 47 patients, LC was performed using the D-line approach (DLC group), and in the consecutive 65 patients, the conventional approach (CLC group) was used. The Ethics Committee and Institutional Review Board of Alexandria Faculty of Medicine approved the study, which was conducted in accordance with the Declaration of Helsinki. Written informed consent was obtained from all patients involved in the study.

Surgical technique

Laparoscopic cholecystectomy was performed in all patients using the conventional four-port technique. The pneumoperitoneum was created either through the open or closed method. A 30° laparoscope was introduced through the first 12 mm port which was inserted in the umbilical scar or above the umbilicus. A second 12 mm working port was inserted in the epigastric region, two fingers below the xiphoid process and to the right of the midline and the falciform ligament at the inferior liver border. The other two 5 mm ports were inserted in the subcostal area along the mid-clavicular line and anterior axillary line.

Conventional approach

In the conventional method, the imaginary line extending from the base of liver segment IV to the RS was used as an anatomical landmark above which dissection of the GB was performed. The CVS was achieved by first incising the posterior peritoneal layer of the lower part of the GB, moving through the anterior peritoneal layer, and then dissecting the cyst duct, cystic artery, and the neck of the GB.

D-line approach

**Figure 1:**
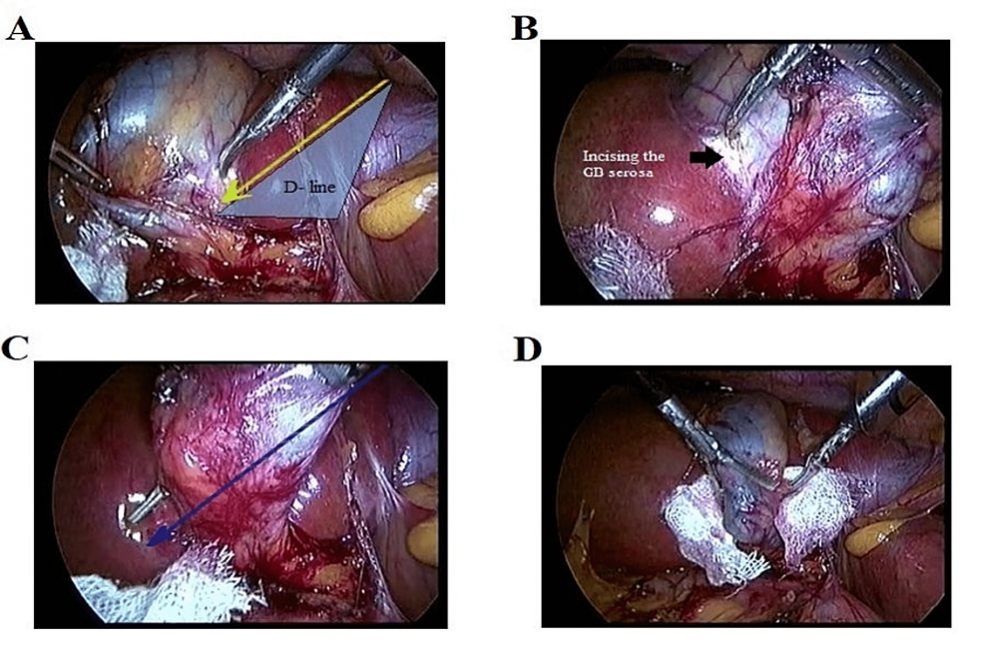
A. Incising the anterior GB serosa along the D-line of liver segment IV. B. Incising the posterior GB serosa along the D-line. C. The lower part of GB is successfully isolated. D. A surgical gauze is inserted behind the lower part of GB along the D-line. GB: gallbladder

**Figure 2:**
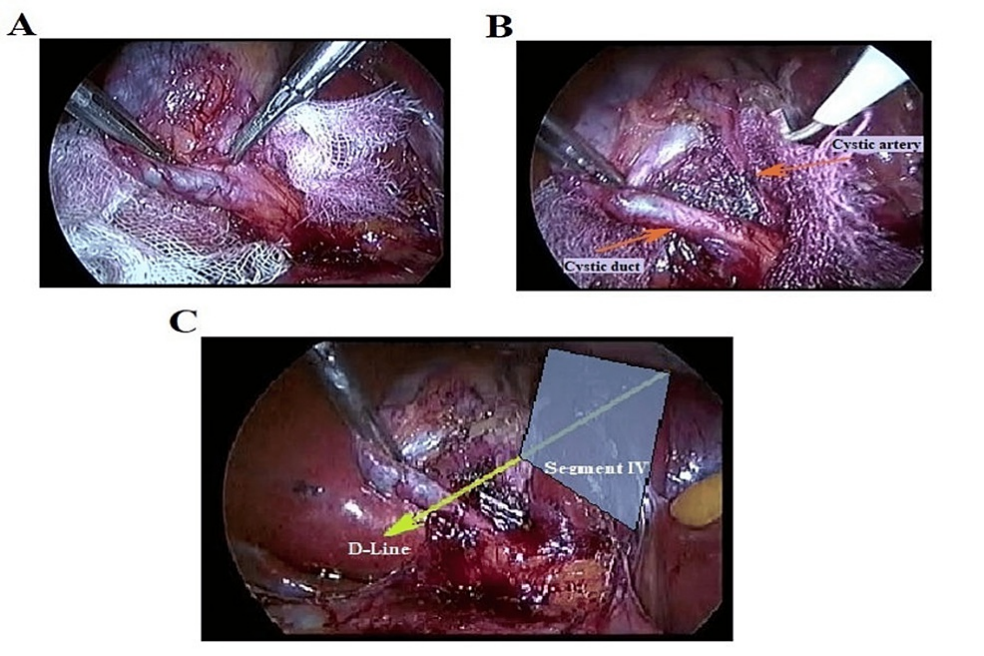
A. Dissection of cystic structures in front of the surgical gauze. B. Achievement of critical view of safety. C. The plane along the D-line is away from any vasculo-biliary structures.

**Figure 3:**
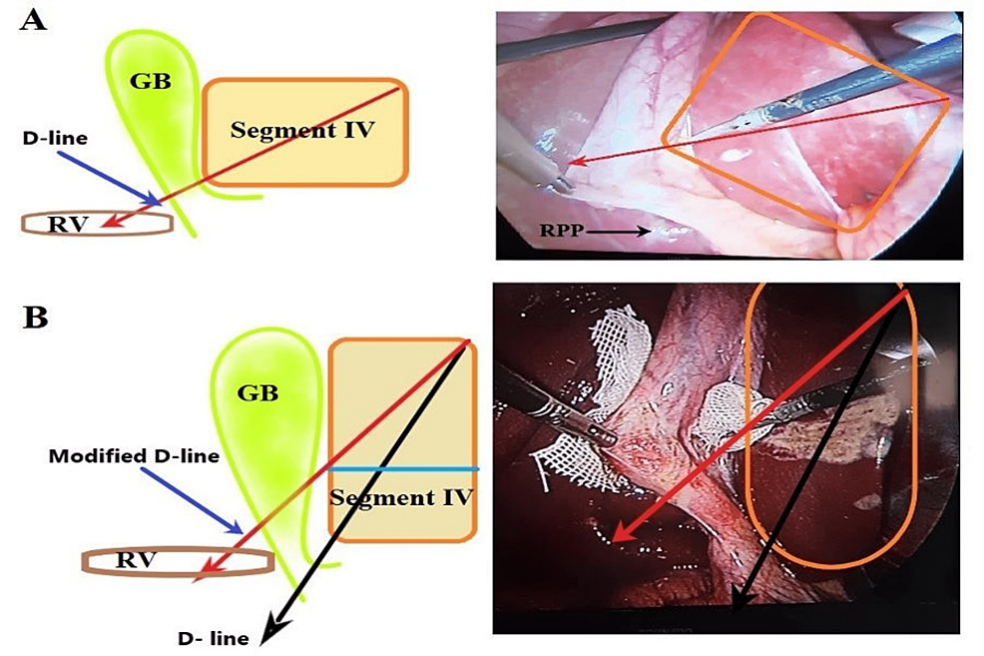
Diagram and intraoperative photos of the inferior surface of segment IV and its relation to the imaginary D-line. (A) Segment IV had an almost square or horizontally rectangular shape (mediolateral dimensions were the same or longer than anteroposterior dimensions). (B) Segment IV's inferior surface had a vertical rectangular shape (the anteroposterior dimension was longer than the mediolateral dimension) and the imaginary D-line passed below the RS. D-line: the diagonal line of Segment IV; GB: gallbladder; RPP: right posterior pedicle; RV/RS: Rouviere’s sulcus.

On the other hand, the D-line approach has been used but with some modifications to the previously described technique [1,7]. The falciform ligament was not tied or retracted extracorporeally because the cephalad retraction of the GB was enough to expose the inferior surface of segment IV. Initially, the lower third of the GB was dissected from the liver bed along the imaginary D-line of the liver segment IV (Figure 1A, 1B) through the subserosal layer of the GB wall, starting anteriorly, then posteriorly above the level of RS or the right posterior pedicle (RPP) [8] to make a window behind the GB through which a surgical gauze was inserted to serve as a landmark (Figure 1C, 1D). In most cases, Maryland dissecting forceps were used, and sometimes the Harmonic scalpel was used for the dissection of the lower third of GB. In front of that gauze, cystic structures were dissected, and fat and fibrous tissues were cleared off the Calot's triangle (Figure 2A, 2B), then the gauze was removed, and CVS was achieved (Figure 2C). When LC was difficult due to severe scarring in the Calot's triangle, further dissection of the cystic structures was stopped after making the window behind the GB, and a subtotal cholecystectomy was done at the level of the D-line. In some cases, after cephalad retraction of the GB, the shape or geometry of segment IV's inferior surface was vertically rectangular (the anteroposterior dimension was longer than the mediolateral dimension) and the imaginary D-line passed below the RS as demonstrated in Figure 3 [9]. Therefore, a modified D-line was used in those patients running along the junction between the upper two-thirds and lower one-third of the GB anteriorly and above the RS or RPP, in the absence of RS, posteriorly (Figure 3).

Statistical analysis

In this study, the sample size was calculated using G*power software version 3.0.10 using the following parameters: α = 0.05, power = 80%, and difference in means (δ) of operative time based on a previous study [10], 25 patients or more should be included in each group. For statistical analysis, we used the Statistical Package for the Social Sciences (SPSS, version 25.0; IBM Corp., Armonk, USA). A chi-square test or Fisher's exact test was used to compare categorical variables. In contrast, continuous variables were represented as medians and interquartile ranges and compared with the Mann-Whitney U-test. P-values less than 0.05 were considered statistically significant.

## Results

The preoperative characteristics of patients of both groups were summarized in Table 1. There were no significant differences regarding age, body mass index (BMI), and the preoperative laboratory parameters. The ultrasonographic findings of the GB, such as the presence of a thickened wall of GB, impacted stone in the GB neck, GB stone > 2.5 cm, or contracted GB, were comparable between both groups. Because of the severe fibrosis and sclerosis in the Calot’s triangle, three patients underwent subtotal cholecystectomy in the DLC group along the D-line. No patients underwent subtotal cholecystectomy in the other group (p= 0.07) (Table 2). In the CLC group, four patients converted to open surgery due to unclear biliary anatomy and bleeding from the liver bed, while in the DLC group, a cystic artery injury occurred in one patient during the dissection of a severely sclerotic Calot's triangle leading to obstruction of the field of vision, which prompted the conversion to an open cholecystectomy to control bleeding and prevent further BDI (p=0.40). There was no significant difference between both groups concerning the intraoperative findings, such as the presence of adhesions or the ease of dissection of the Calot’s triangle and the GB from the liver bed, as shown in Table 2. Furthermore, the operative time and intraoperative blood loss were comparable between both groups (p=0.28, p=0.66, respectively).

**Table 1:** Perioperative characteristics of patients of both groups. ALP: alkaline phosphatase; ALT: alanine transaminase; AST: aspartate transaminase; BMI: body mass index; CBD: common bile duct; GB: gallbladder; Hb: hemoglobin; HBV: hepatitis B virus; HCV: hepatitis C virus; INR: international normalized ratio; TB: total bilirubin; WBCs: white blood cells; DLC: diagonal line approach laparoscopic cholecystectomy; CLC: conventional laparoscopic cholecystectomy.

		DLC Group (n=47)	CLC Group (n= 65)	p
Age (years)		39 (31-51)	40 (32-52)	0.53
BMI (kg/m^2^)		33 (23-39)	34 (26.5-38.5)	0.85
Preoperative laboratory parameters	Hb (g/dl)	12.9 (12.1-13.3)	12.4 (11.9-13.3)	0.15
	WBCs (x 10^9^/L)	6.9 (5.5-8.8)	7.3 (5.8-8.9)	0.44
	INR	0.99 (0.97-1.03)	0.99 (0.96-1.06)	0.90
	TB (mg/dL)	0.5 (0.3-0.63)	0.4 (0.23-0.5)	0.12
	ALP (U/L)	74 (60-94)	81 (63-104)	0.30
	ALT (U/L)	23 (18-31)	22 (18-31)	0.64
Preoperative ultrasound of GB (n, %)	Thickened wall (> 4mm)	14 (30)	30 (46)	0.11
	Contracted GB	10 (21)	7 (11)	0.18
	Impacted stone in the GB neck	4 (8)	8 (12)	0.75
	GB stones > 2.5 cm	4 (8)	5 (8)	1
	Multiple GB stones	39 (83)	44 (68)	0.08

**Table 2:** The intraoperative findings in both groups. DLC: diagonal line approach laparoscopic cholecystectomy; CLC: conventional laparoscopic cholecystectomy.

			DLC Group (n=47)	CLC Group (n=65)	p
Operative parameters	Operative time (min)		40 (30-50)	40 (40-50)	0.28
	Blood loss (ml)		30 (10-50)	30 (20-50)	0.66
	Conversion to open (n, %)		1 (2)	4 (6)	0.40
	Conversion to subtotal cholecystectomy (n, %)		3 (6)	0	0.07
Gallbladder adhesion (n, %)	None		20 (43)	36 (55)	0.1
	Partial		17 (36)	25 (38)	
	Complete		9 (19)	3 (5)	
	Dense		1 (2)	1 (2)	
Dissection of Calot’s triangle (n, %)		Clear anatomy	24 (51)	32 (49)	0.33
		Fat covers anatomic structures	18 (38)	31 (48)	
		Unclear anatomy	4 (9)	2 (3)	
		Anatomic variations	1 (2)	0	
Dissection of Gallbladder (n, %)		Easy	34 (72)	46 (71)	0.12
		Adherent to the liver	11 (24)	8 (12)	
		Edema of the wall	1 (2)	6 (9)	
		Fibrosis of the wall	1 (2)	5 (8)	

## Discussion

Despite recent advances in surgical instruments and techniques, BDI is still present at a rate of 0.4-0.7% in LC [11], whereas its incidence after open cholecystectomy is around 0.2%. The CVS implementation did not significantly decrease the incidence of BDI in LC, and some authors even reported that BDI occurred during its implementation [12-15]. One of the drawbacks of the CVS concept is that it simply describes the final key findings before clipping and cutting of cystic duct and artery, without showing how to accomplish these, like showing the correct door out of a labyrinth, but without illustrating how to get there [13,14,16]. That is why some authors recommended the use of anatomical landmarks as a guide during the dissection of Calot’s triangle [5,12,17]. Honda et al [13] proposed dissection of the GB along the inner layer of the subserosa of the GB wall above the level of the RS to avoid BDI. Whereas Tokyo guidelines in 2018 recommended dissection of the Calot’s triangle and GB during LC above an imaginary line between the base of liver segment IV and the RS [5]. Nevertheless, RS is present in only 75% of patients and sometimes the base of segment IV is obscured by inflammation or adhesions [1,18]. Furthermore, It is also possible that the CBD can be above RS in cases of excessive upward traction of Hartmann's pouch or shrunken and fibrosed GB [17]. Here comes the strength of the D-line technique which depends on segment IV as a whole which is always visible in all patients [9]. Segment IV's diagonal line generally indicates the location between the GB and cystic plate in which there are no significant vascular or biliary structures. So, it is absolutely safe to dissect in this area regardless of the presence of any vascular or biliary anomalies, or severe inflammation in the Calot's triangle [1]. Fujioka et al [7] used the D-line approach for LC in patients with bile duct anomalies and found that it was safe and very effective in avoiding BDIs. The D-line served as a landmark to help surgeons dissect Calot's triangle to the right side and away from the hilar plate, which is known to be the convergence point for all biliary ducts, including the anomalous bile duct [7]. Furthermore, Kitamura et al [1], in their study on 62 difficult LCs, successfully performed total cholecystectomy on 71% of patients using the D-line approach without any significant postoperative complications.

Many studies suggest that bail-out procedures like laparoscopic subtotal cholecystectomy, fundus first cholecystectomy, or open conversion should be performed in difficult LCs in which the CVS cannot be achieved due to severe sclerosis or fibrosis or unclear anatomy to prevent BDI [5,16]. For laparoscopic subtotal cholecystectomy, the D-line approach has been found to be one of the most useful techniques because the initial step includes separating the lower third of the GB from the liver bed along the D-line. Hence, in case of severe sclerosis or unclear anatomy in the Calot's triangle, the dissection was not further performed, and subtotal cholecystectomy was performed at the level of the D-line [1]. The fundus-first cholecystectomy is one of the bailout techniques used when the dissection of the Calot's triangle is difficult. However, some studies have reported severe vasculobiliary injuries following this procedure due to severe inflammation and the wrong plane of dissection of the GB behind the cystic plate, resulting in injury to the right hepatic pedicle [5,19,20]. Hence, some authors proposed novel techniques of laparoscopic fundus first subtotal cholecystectomy in cases of difficult LC. An example of this is the study performed by Harilingam et al [21], in which the fundus was opened and the GB was split, followed by the anterior wall being removed and the posterior wall cauterized. They used the inner lumen of the GB as a landmark to avoid vasculobiliary injury during cholecystectomy. Nasr [22] used an anatomical point which was the joining of the cystic artery and GB distal infundibulum as an anatomical landmark during fundus-first subtotal cholecystectomy to avoid serious vascular or biliary injury. This anatomical landmark, however, was vague and not always obvious, especially in difficult cases with severe adhesions. The D-line approach, on the other hand, is a more reliable and acceptable landmark for laparoscopic fundus first subtotal cholecystectomy if the LC is difficult, with severe adhesions and the CVS cannot be achieved.

## Conclusions

In conclusion, the D-line approach provides a step-by-step dissection of Calot’s triangle and the GB from the liver bed to achieve the critical view of safety or to perform subtotal cholecystectomy along the D-line in case of severe sclerosis and fibrosis of Calot’s triangle. Nevertheless, more studies, especially randomized studies, are required to further assess this technique and to determine if it is superior to the conventional technique or not. This study is limited by its non-randomized nature and the small number of patients; however, it is the first study to externally validate the D-line approach.
